# A Treatise on the Nature and Cure of Gout; Comprehending a General View of a Morbid State of the Digestive Organs, and of Regimen: With Some Observations on Rheumatism

**Published:** 1817-12

**Authors:** 


*A Treatise on the Nature and Cure of Gout; comprehending a general View of a morbid State of the Digestive Organs, and of Regimen: with some Observations on Rheumatism.* By CHARLES SCUDAMORE, M. D. Member of the Royal College of Physicians, &c. &c. 8vo. pp. 402. London, 1816.

IF we have been tardy in noticing the work of Dr. Scudamore on Gout, the omission, that gentleman may rest assured, has arisen from no feeling of disrespect towards himself, and no inability or unwillingness to appreciate aright the importance of his labours. Indeed, there are no men who feel more warmly interested in the reputation and success of Dr. Scudamore, than the writers of the Medico-Chirurgical Journal; or who more highly estimate the value of a production, which alike confers honour upon its author, and on the science with which it is connected. The particular origin of such sentiments towards himself, Dr. Scudamore will, doubtless, be at no loss to comprehend.

It were needless to specify here the causes from which arose our delay in reviewing this publication. Alloyed with no contemptible or unworthy motive, they are such as Dr. Scudamore, if he knew them, would respect. But they now no longer exist: and we are unwilling that another year should close upon our labours without some notice of the work, and some vindication of ourselves from the charge of neglect, which, otherwise, might be brought against us. Yet, as a second and enlarged edition of the Treatise has just issued from the press, it will obviously be proper to make that the subject of our criticism: and this we propose to do in our succeeding Number. The alterations and improvements in the last edition will be carefully noted.

